# General practice needs further development of theoretical knowledge

**DOI:** 10.1080/02813432.2019.1641900

**Published:** 2019-07-16

**Authors:** Geir Sverre Braut, Svein R. Kjosavik

**Affiliations:** a The General Practice and Care Coordination Research Group, Stavanger University Hospital, Stavanger, Norway;; b SEROS – Centre for Risk Management and Societal Safety, University of Stavanger, Stavanger, Norway;; c Faculty of Health Sciences, University of Stavanger, Stavanger, Norway

Per Fugelli stated his imperative for general practice in 1984: You must write [about] your subject! [1]. He was concerned about the lack of scientific documentation and research in our discipline, and emphasized the need of knowledge and theoretical understanding of the unique distinctive character of general medicine. In 2017, Guri Rørtveit concluded that primary care research in Norway was going in the right direction [2]. The extent of research, the number of researchers and funding of general medical research has increased considerably.

Nevertheless, there has been a change in medical practice that challenges primary health care fundamentally. The responsibility for people with a complex set of diseases is increasingly transferred to GPs. Consequently, there is a need for more research-based knowledge and a broader theoretical basis for embracing the current challenges, and to influence the further developments of medicine in general and primary health care in particular.

Back in 1962 Thomas S. Kuhn published his book on the structures of scientific revolutions [3]. There he claims that scientific development does not always evolve gradually. From time to time the contemporary theories for explaining empirical data turn out to be insufficient. A need for radically new theories emerges, as the current theories collapse. Even though heavily debated, the work of Kuhn has had great impact in some scientific fields, as for example economy and humanities. In medicine and the natural sciences, his thoughts have left fewer traces.

The theoretical basis for research methods in medicine is to a high degree based upon hypothetical deductive approaches, compatible with Karl Popper’s falsifiability criterion [4]. No doubt that this has led to, and still leads to, great advances in medical knowledge, producing results that are applicable in clinical work.

From evidence-based medicine we have learned to regard the double blind randomized trial as the gold standard for testing, and eventually refuting, a possible cause-effect relationship. This approach has its strength when dealing with causal chains that can be understood and modelled as linear interactions [5]. When studying the effects of single pharmaceuticals or evaluating possible cause for diseases that can be clearly isolated, this is a sensible approach.

In a medical perspective, such a predominantly monocausal approach to challenges in medical research, has its roots over 150 years back. At that time, models of this type laid the foundation for the immense development in bacteriology as well as in other branches of medicine. It has taught us how to sort out the effective means from those being useless.

Today there seems to be an increasing doubt about the feasibility of monocausal ways of dealing with clinical problems on an individual level [6]. Meeting the current challenges in clinical medicine, often with a possible complex pathogenesis, e.g. multimorbidity, chronic fatigue, psychotic disorder and, not least, upcoming technologies related to individualized medicine, we need to expand our understanding of what should be considered as valid knowledge.

The core of this discussion concerns how to consider uncertainty. When approaching populations, it is sensible, at least partly, to model uncertainty in terms of probability. The underlying presumption is that it is possible to study a sequence of random trials that approaches infinity [7]. When meeting single patients neither is it possible to obey the principle of randomness, nor the principle of infinity.

Some years back Grossi suggested that the concept of probability could be substituted by plausibility when discussing individual risk [8]. In this way, the clinician could use stochastic information from quantitative studies in combination with general medical and specific patient related knowledge when reflecting upon relevant clinical actions. In general practice, we assume that the clinician is thinking more in line with plausibility for a desired effect in a specific situation, than probability based upon generalized knowledge, when, for example, deciding to use or not to use antibiotics for treatment of an infection in the upper airways in a certain patient. This way of approaching the concept of risk is in accordance with current developments in safety science [9].

If sticking to a development in this direction we, at least when treating individual patients, have to abandon the widely accepted understanding of risk as the probability for an event to occur [7]. Risk then would be a more complex concept, a combination of the set of possible outcomes with the uncertainties connected to them [9]. The uncertainties are of a probabilistic (aleatoric uncertainty) nature as well as related to the extent and quality of knowledge available (epistemic uncertainty).

Perhaps Simpkin and Schwartzstein hit the nail on the head when they ask weather tolerating uncertainty will be the next medical revolution [10]. They demonstrates that this will require a conceptual shift in theoretical thinking as well as practice in medicine, e.g. speaking of questionable “hypotheses” rather than striving for exact “diagnoses” in the clinical communication around single patients. This way of thinking and working is often well integrated in general practice.

Finding exact and cause specific diagnoses, seems to define a golden standard for medical practice. When approaching diseases that can be described and understood according to a principle of one agent, one disease, this seems to be a sound theoretical platform. But when trying to tackle diseases with causal complexity [11], a monocausal approach may be suboptimal or even directly dangerous to the patient.

Medicine as a practice and science dealing with uncertainty still seems to be regarded more like a possible imperfection or deviation from this standard. Two of the distinctive features related to general practice are the ability to meet the patient in her or his context, and to follow up over a period of time. Combining these features with a novel understanding of the concepts of uncertainty and risk, could trig development of a broader theoretical platform for medicine than we tend to acknowledge today.

Perhaps it is not worth calling it a scientific revolution, as other sciences already are well on their way down this road. But a wider perspective on multicausality, accepting that diseases not least seen in general practice often are related to a complex of causal factors, could provide medicine with new theories, research methods and clinical practices. A broad and systematic view on uncertainty then will be a core issue.
